# Influence of Storage Conditions and Preservatives on Metabolite Fingerprints in Urine

**DOI:** 10.3390/metabo9100203

**Published:** 2019-09-27

**Authors:** Xinchen Wang, Haiwei Gu, Susana A. Palma-Duran, Andres Fierro, Paniz Jasbi, Xiaojian Shi, William Bresette, Natasha Tasevska

**Affiliations:** 1 Jiangxi Key Laboratory for Mass Spectrometry and Instrumentation, East China University of Technology, Nanchang 330013, China; xwang547@asu.edu; 2 College of Health Solutions, Arizona State University, Phoenix, AZ 85004, USA; haiweigu@asu.edu (H.G.); sussypalma@hotmail.com (S.A.P.-D.); andres79@asu.edu (A.F.); pjasbi@asu.edu (P.J.); xshi49@asu.edu (X.S.); wbresett@asu.edu (W.B.)

**Keywords:** temperature, preservative, mass spectrometry, metabolomics, urine storage

## Abstract

Human urine, which is rich in metabolites, provides valuable approaches for biomarker measurement. Maintaining the stability of metabolites in urine is critical for accurate and reliable research results and subsequent interpretation. In this study, the effect of storage temperature (4, 22, and 40 °C), storage time (24 and 48 h), and use of preservatives (boric acid (BA), thymol) and para-aminobenzoic acid (PABA) on urinary metabolites in the pooled urine samples from 20 participants was systematically investigated using large-scale targeted liquid chromatography tandem mass spectrometry (LC-MS/MS)-based metabolomics. Statistical analysis of 158 reliably detected metabolites showed that metabolites in urine with no preservative remained stable at 4 °C for 24 and 48 h as well as at 22 °C for 24 h, but significant metabolite differences were observed in urine stored at 22 °C for 48 h and at 40 °C. The mere addition of BA caused metabolite changes. Thymol was observed to be effective in maintaining metabolite stability in urine in all the conditions designed, most likely due to the inhibitory effect of thymol on urine microbiota. Our results provide valuable urine preservation guidance during sample storage, which is essential for obtaining reliable, accurate, and reproducible analytical results from urine samples.

## 1. Introduction

Metabolomics is a field concerned with the comprehensive monitoring of thousands of metabolites (mass < 2000 Da) in biological systems for assessing metabolic modifications caused by physiological/environmental factors at the molecular level [1,2,3,4,5]. Among various biological matrices used in metabolomics, urine is a non-invasive biofluid that carries abundant water-soluble metabolic waste products. Recent studies have identified more than 3000 metabolites in urine [6]. Due to the appealing advantages of non-invasive sample collection, ready availability, and richness of information that urine samples provide, urinary tests are performed widely and extensively for the detection of illegal drug use [7,8], renal function monitoring [9,10], diagnosis [11,12,13], cancer risk assessment [14,15], disease biomarker identification [16,17,18], etc., and provide valuable insights into human health and disease pathogenesis. Metabolite signatures in urine can also be used as biomarkers of dietary patterns or intake of specific foods, and may also help elucidate mechanistic pathways relating diet to chronic disease risk [19]. Therefore, urine plays an important role in metabolomics and metabolism research.

However, urinary metabolites can be easily affected and changed by factors such as temperature, storage duration, and contamination by bacteria, which may contribute to misleading results. To minimize urinary metabolite changes, it is of significant interest and importance to use proper urine collection and storage conditions to ensure the integrity of metabolites and fidelity of results. Improper urine storage conditions, such as exposure to high temperature or long-term transportation, may lead to water evaporation [20], bacterial growth and/or contamination [21], and potentially significant changes in urine composition. Although urine of healthy subjects is expected to be sterile, normal bacterial flora present in the distal part of the urethra can contaminate urine [22]. There is an urgent call by the community for careful review and standardization of the preanalytical process. Previous studies have demonstrated the effects of storage temperature, preservative use, and storage time on metabolite profiles [23,24,25]. Boric acid (BA) [26], a weak acid used as a mild antiseptic in agriculture and medicine, is a preservative of choice in 24 h urine collection protocols for measurement of dietary biomarkers [27,28,29,30]. Thymol [31] is a natural monoterpenoid phenol derivative of cymene and is widely used as a flavoring agent. Diluted in isopropanol, thymol has been used as a disinfectant and preservative. However, due to its flammability, it has had limited use in population and clinical research. Bacterial overgrowth can be prevented effectively with other preservatives such as sodium azide [32] (10 mM), which is highly toxic, as well as chlorhexidine [33] (0.4 mM). So far, few studies have used targeted metabolomics, which has the advantages of enhanced detection sensitivity and specificity, to systematically investigate the influence of storage temperature, storage duration, and use of preservatives on urinary metabolites [25,34]. Yet, no study has thus far investigated the effectiveness of preservation methods for urine storage at high temperatures, which simulate summer outdoor temperatures in hot climates.

Our aim is to develop an appropriate protocol for 24 h urine collection that will ensure stability of metabolite signatures as potential biomarkers of diet and disease. In this study, we systematically investigated the effects of three temperatures (4, 22, and 40 °C), two storage durations (24 and 48 h), and three preservative methods (No preservative (NP), boric acid, and thymol) (Table 1) on the stability of metabolite signatures in urine with or without para-aminobenzoic acid (PABA), a compound that is commonly used as a marker of 24 h urine completeness in dietary biomarker studies [35], using a highly sensitive liquid chromatography tandem mass spectrometry (LC-MS/MS)-based metabolomics approach. To the best of our knowledge, this is the first study to investigate preservation methods at high temperature.

## 2. Results

### 2.1. Effects of Temperature and Duration of Storage on Urine Metabolites

**Figure 1:**
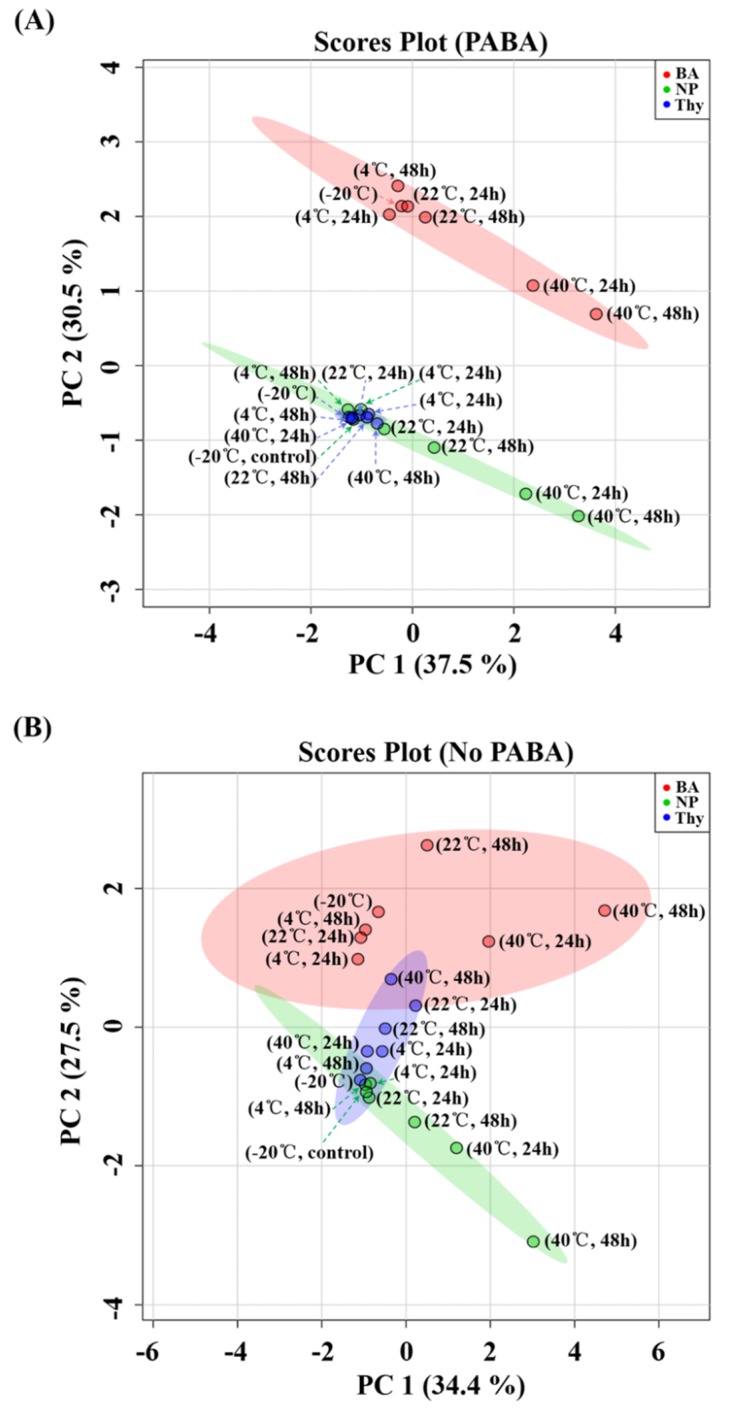
Principal component analysis (PCA) score plot of urine samples in (**A**) PABA group, and (**B**) No PABA group. Different colors represent different preservatives: green (no preservative), red (boric acid), and blue (thymol).

The influence of low temperature (4 °C), room temperature (22 °C), and high temperature (40 °C) settings with 24 and 48 h storage durations on urinary metabolites was studied. Urine samples with NP stored at −20 °C were used as controls for analytical purposes. For the NP samples, the principal component analysis (PCA) score plots showed that controls (−20 °C) and 4 °C samples were clustered together, indicating that urine metabolites remained stable at 4 °C in both PABA (Figure 1A) and no PABA (Figure 1B) pooled samples, regardless of storage duration (24 or 48 h). This indicates that urine containing no preservative can maintain the integrity of metabolite signatures at refrigeration temperatures (4 °C) for up to 2 days (Table 2).

At room temperature (22 °C), the NP samples stored for 24 h were clustered with controls in both PABA (Figure 1A) and No PABA (Figure 1B) groups. However, when the storage period was extended to 48 h, the NP samples stored at 22 °C for 48 h were situated farther apart from controls in the PCA score plot, demonstrating that at room temperature (22 °C), urine can only be stored for 24 h but not 48 h. All the NP 40 °C samples were located far away from NP controls, indicating that high temperature (40 °C) strongly affected the integrity of urine metabolites, regardless of storage period.

### 2.2. Effects of Different Preservatives on Urine Metabolites

The effects of preservatives on metabolite changes in urine were evaluated. As shown by the PCA score plot (Figure 1), urine samples treated with BA stored at −20 and 4 °C were clearly separated from controls in both PABA and No PABA groups. In the PABA group, the BA 22 °C 48 h samples were clustered with BA −20 and 4 °C samples. In the No PABA group, the BA samples stored at 22 °C for 48 h were separated from BA-treated samples stored at −20 and 4 °C. Additionally, a significant separation between BA-treated samples stored at 40 °C and BA-treated samples stored at −20 and 4 °C was observed. Supplementary Figure S2 shows typical overlapped extracted ion chromatograms of the detected metabolites. Urine samples stored with thymol at all temperatures (−20, 4, and 40 °C) at each time point (24 and 48 h) were clustered with controls in both PABA and No PABA groups (Figure 1), compared with urine samples with no preservative stored at −20 °C, 4 °C for 24/48 h, and at 22 °C for 24 h.

In the PCA score plot of the PABA group (Figure 1A), urine samples with BA were distinct in PC scores from those of thymol or no preservative, but no such distinction could be made between the latter two groups. Nonetheless, thymol scores generated a much narrower 95% confidence region than untreated samples (NP), indicating less metabolite changes in Thy samples as compared to NP and BA. In the PCA score plot of the No PABA group, urine samples treated with thymol did not display the same degree of clustering as the samples from subjects given PABA; subsequently, a much wider 95% confidence region was observed.

**Figure 2:**
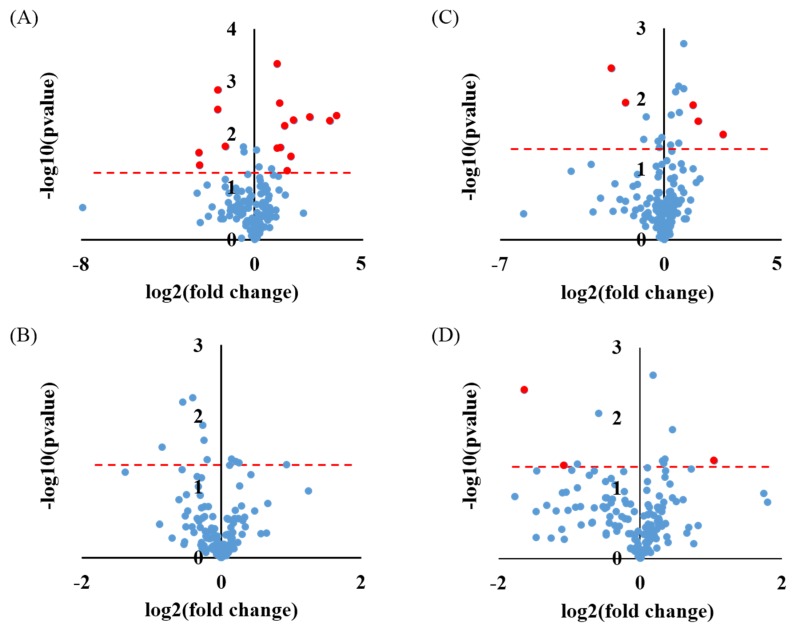
Volcano plots of low temperature (−20 °C, 4 °C/24 h, 4 °C/48 h) versus high temperature (40 °C/24 h, 40 °C/48 h) in the PABA group with (**A**) no preservative, (**B**) thymol and in the No PABA group with (**C**) no preservative, and (**D**) thymol. Red dots indicate metabolites with *p*-value < 0.05 and fold change (low temperature (−20 °C, 4 °C)/high temperature (40 °C)) > 2 or < 0.5.

The volcano plots were plotted in Figure 2 to obtain the significantly altered metabolites between −20/4 °C and 40 °C samples with NP or thymol. In both the PABA and No PABA groups, NP samples at 40 °C showed greater significance (more red dots) and a higher degree of change than Thy samples at 40 °C (Figure 2). Interestingly, no significant metabolites (no red dots) with an appreciable amount of change were observed in the PABA Thy group (Figure 2B). Table 3 and Table 4 list the significantly altered metabolites with *p* < 0.05 and fold change (FC, (−20 °C, 4 °C)/(40 °C)) > 2 or < 0.5 (shown as red dots in Figure 2). A comparison between metabolite profiles of urine with thymol and no preservative stored at −20 °C, 4 °C for 24/48 h, and at 22 °C for 24 h showed nine and three out of 158 metabolites significantly altered in the PABA and No PABA group, respectively (Supplementary Figure S1B,D and Table S1).

## 3. Discussion

Our experimental results showed that low temperature storage (4 °C) of urine samples can minimize metabolite changes in urine, which is consistent with results by Roux et al. [25], whereas high temperature (40 °C) can easily result in metabolite changes. Interestingly, when urine samples were stored at room temperature (22 °C), there was no significant change in metabolites within 24 h, but significant changes occurred in samples stored for 48 h. Additionally, the effects of storage temperature are moderated by storage duration. For short-term storage (hours), refrigeration at 4 °C was shown to be sufficient to inhibit metabolite degradation [25,36]. For longer storage time (weeks or months), temperature of −20 or −80 °C is preferred [23,37]. For specimen repositories that demand storage periods spanning years [38], cryopreservation in liquid nitrogen at −180 °C is required. 

Laparre et al. [23] set up a series of low temperature conditions to explore the effects of long-term storage of urine (>30 days). Their subsequent observations also underscored the importance of determining storage duration before designating a specific storage temperature (e.g., −80 °C for long-term storage). Rotter et al. [24] used targeted MS (63 monitored metabolites) to investigate the integrity of urine samples under different storage temperatures (−80, −20, 4, 9, and 20 °C) and storage durations (0, 2, 8, and 24 h). They found that only seven out of 63 investigated metabolites, mainly amino acids, were altered when urine was stored at 9 and 20 °C for 24 h but not 2 or 8 h, and detected a statistically significant interaction between storage duration and storage temperature on metabolite concentrations. Our experimental results consistently demonstrated this interaction as evidenced by significant metabolite changes between urine samples stored at room temperature for 24 and 48 h. Dunn et al. [39] coupled GC-TOF-MS data (>700 metabolite peaks) with statistical analysis (PCA) to assess the influence of storing urine at 4 °C over 0 or 24 h versus being frozen at −80 °C. Similarly to ours and other studies [23,24,25], they found that storing urine samples at 4 °C for up to 24 h is suitable for maintaining metabolite stability in metabolomic studies.

The addition of preservatives to urine samples is commonly used to prevent decomposition and decay during long-term and/or high-temperature storage. Eisinger et al. [36] found that preservative-containing BD (Franklin Lakes, NJ, USA) vacutainer collection tubes were desirable for urine samples at room temperature. Lauridsen et al. [37] concluded that addition of preservatives or storage at temperatures below −25 °C was necessary for urine samples if sterility could not be assured. Meanwhile, specific urine preservation methods can be employed for different analytes of interest. For example, Hoppin et al. [40] suggested that no refrigeration or preservative use was needed for measurement of environmental contaminants present in urine. In contrast, Thierauf et al. [33] found that refrigerated storage conditions or preservatives were necessary to inhibit the degradation of analytes such as ethyl-glucuronide and ethyl-sulphate. Use of analyte-specific preservation techniques is also suggested by the results of this study (Table 3 and Table 4).

There are literature and standards shared by the community, which call for more strict sample collection and storage conditions to avoid drifts in the metabolic fingerprint [25,34,41,42]. Such considerations include the experiences of both nuclear magnetic resonance spectroscopy (NMR) and MS analysis [3,34]. Urinary preservation methods have been assessed by monitoring multiple metabolites in parallel. Roux et al. [25] used high resolution-MS (280 identified metabolites) to study the impact of sample collection conditions (room temperature, 4 °C, with or without BA as the preservative, stored up to 72 h) on the metabolite composition of human urine. They found that the presence of BA (200 mM) regardless of temperature and duration of storage affected the metabolic composition of urine. Similarly, our results showed that BA prevented bacterial growth but still formed a distinct cluster on the PCA score plot. Interestingly, the effect of BA on urinary metabolites might be closely related to the concentration of BA. To further investigate the influence of BA on metabolites in urine, Xiao et al. [34] used gradient concentrations of BA and found that the addition of 20 and 200 mM BA would cause significant separation between BA groups and a control group, and that 2 mM BA would be suitable for urinary metabolomics research. We found that the addition of BA in concentration needed for preservation of biomarker of intake (2 g/L; i.e., 32.3 mmol/L) [27,28,29,30] changed the metabolic profile of urine in both PABA and No PABA groups. Yet, when we compared metabolite profiles of BA samples with NP stored at −20 °C, 4 °C for 24/48 h, and at 22 °C for 24 h, only few metabolites were significantly altered (Supplementary Figure S1A,C and Table S1), indicating that BA may still be a good choice for use as a preservative in urine-based metabolomics studies. BA was used for NMR-based metabolomics studies [41,42] because only minor distinctions were detected between samples with and without BA. However, with higher analytical sensitivity of MS-based metabolomics methods, the effect of BA on metabolites in urine was revealed by Roux et al. [25], Xiao et al. [34], and us. It may be that BA alters the ionization properties of the metabolites in LC-MS-based studies as shown by Rebane and Herodes [43] who reported significant changes in signal intensities in LC-ESI-MS after adding BA. The observed separation in the PCA score plot (Figure 1B) between the BA 22 °C 48 h and BA −20/4 °C samples in the No PABA group showed that BA presented limited preservative efficacy in urine stored at 22 °C for ≥48 h, while the separation between BA 40 °C and BA −20/4 °C samples (Figure 1A,B) showed that BA had no preservative efficacy at 40 °C.

It is worth noting that Table 3 showed that several carbohydrates (sucrose, trehalose, lactose, and fructose) were significantly decreased when the urine was stored at 40 °C without preservatives, which indicated that these carbohydrates may have served as substrates for microorganisms. The clustering of Thy −20/4 °C samples and NP −20/4 °C samples indicated that the addition of thymol did not strongly interfere with metabolite composition and only minor metabolite changes were detected for Thy samples at 22 °C/48 h or even high temperature (40 °C) storage conditions. It is worth noting that thymol was the only preservative to show minor variation at 40 °C (Figure 1), indicating that thymol was effective at preserving urine samples even at high temperature.

The purpose of volcano plots was to obtain the significantly changed metabolites under 40 °C storage conditions with and without preservatives. Given that thymol was the only preservative found to be effective under 40 °C storage conditions (Figure 1), only thymol was included for significance testing. As evidenced by a fewer number of significantly changed metabolites with considerable fold changes (Figure 2), thymol was found to be superior preservative for urine storage at high temperatures (e.g., 40 °C). Some amino acids (tyrosine, isoleucine, and methionine) were easily affected, which was consistent with the results by Rotter et al. [24]. The results of our PCA analysis (Figure 1), significance testing, and fold change analysis (Figure 2, Table 3 and Table 4) clearly demonstrate that a preservative is required for urine storage at high temperature, and that thymol is a good candidate. 

Certain limitations also require further study. For instance, literature has shown interactionary effects between initial metabolite content of cells in urine and observed metabolite changes over time [44,45], underscoring the importance of standardizing specifications for the collection, handling, and preparation of diagnostic samples [46,47]. Given the pooling procedure employed in the current study, possible differences in metabolite intensity may be quenched as a result. Therefore, future studies should evaluate separate samples from each donor in addition to cell content to account for any changes not directly related to storage. Furthermore, we utilized one sample preparation protocol that is frequently used in mass spectrometry-based metabolomics experiments [5,48,49,50]; future studies should monitor possible changes in metabolites using more than one pertinent protocol, as various differences among them could potentially quench differences associated with storage. Additionally, a targeted LC-MS/MS approach [4,24,48], covering ~300 metabolites from >35 metabolic pathways of strong biological significance, such as central carbon metabolism and purine metabolism, was used herein to analyze changes to metabolites with enhanced detection sensitivity and specificity. However, such a targeted approach is inherently limited to metabolites included in the detection panel; future studies should consider analysis using untargeted platforms in order to enhance metabolome coverage.

Compared to previous studies, our study systematically investigated the influence of storage temperature (4, 22, and 40 °C), storage duration (24 and 48 h), and the use of different preservatives (BA, thymol, NP) on metabolite fingerprints in urine using targeted metabolomics (158 reliably detected metabolites). For the first time, the effects of high temperature (40 °C) storage was studied. Our results are in agreement with previous studies [32,51], demonstrating that bacterial contamination can significantly cause numerous biochemical changes in urine. For example, bacteria such as *Staphylococcus* spp., *Streptococcus* spp., *Escherichia coli*, *Klebsiella* spp., *Enterobacter* spp., *Citrobacte*r spp., *Proteus* spp., *Salmonella* spp., *Acinetobacter* spp., and *Pseudomonas* spp. are commonly found in human urine [32] and can metabolize aromatic compounds, resulting in the differential expression of acids in human urine [52].

Informed by these results, we summarize practical guidelines to ensure the integrity of urine samples for future metabolomics studies in Table 5. Urine can easily undergo chemical changes such as oxidation and hydrolysis in open atmosphere. In addition, sugars, amino acids, and proteins contained in urine may help propagate bacterial growth and activity. All these factors can cause degradation of urinary metabolites. In addition, various factors related to storage temperature (lack of refrigeration, being in high-temperature areas), storage time (long-distance transportation, sample collection over weekends), and use of preservatives directly affect metabolites during storage. Our results showed that urine samples can be stored at 4 °C for 48 h, or at 22 °C for 24 h, if no preservative is to be used. If urine needs to be stored at 22 °C for 48 h or in high temperature (40 °C) conditions for 24–48 h, thymol is an effective preservative. While BA prevented bacterial overgrowth in urine stored at 4 and 22 °C for 24 h, it still caused some metabolite changes in urine. However, our results indicate that in a concentration of 2 g/L in urine stored at 22 °C for up to 24 h, BA may still be a good choice as a preservative in urine-based metabolomics studies.

## 4. Materials and Methods 

### 4.1. Study Participants

Twenty male and female participants, >18 years of age, were recruited from students and staff members at Arizona State University (ASU). An email briefly explaining the aims of the study and including a link to the Qualtrics screener for those interested in participating was circulated. Respondents who reported suffering from any sexually transmitted infection, yeast or bacterial infection of the genitourinary tract in the past year, having taken antibiotics or anti-fungal medications within 30 days of the start of the study, having been diagnosed with type 1 or type 2 diabetes, or having an allergy to sunscreen were not eligible to participate. Given that PABA was formerly a common ingredient of sunscreen lotions, an allergy to sunscreen was considered a potential indicator of a PABA allergy. The study was approved by the Institutional Review Board of Arizona State University (ASU IRB # STUDY00002482). All procedures performed in studies involving human participants were in accordance with the ethical standards of the institutional and/or national research committee and with the 1964 Helsinki declaration and its later amendments or comparable ethical standards. Informed consent was obtained from all individual participants included in the study.

Upon consenting, each participant was asked to first empty their bladder prior to consuming a 12 oz sugar-sweetened beverage (Pepsi Throwback, PepsiCo, Purchase, NY, USA) and a sugary snack (three Oreo cookies, Nabisco, Hanover, NJ, USA), provided by the staff at the metabolic kitchen (ASU, Phoenix, AZ, USA). To examine the effect of PABA as a marker of urine completeness, half of the participants (*n* = 10) were asked to take a 100 mg tablet of PABA (Source Naturals, Inc., Scotts valley, CA, USA) with their snacks. 

### 4.2. Urine Sample Collection

Participants were asked to collect a spot urine sample within 2 h of consuming the snacks. They were given 180 mL cups and were asked to start collecting the initial part of the void for maximizing potential contamination of the urine. Immediately after the urine collection, samples from each participant were first split into 12 aliquots of 12 mL each. Four of the aliquots were left untreated (no preservative, NP), four were treated with 24 mg of BA (2 g/L), and four were treated with 60 µL of 10% thymol in isopropanol (5 mL of 10% *w*/*v* solution in isopropanol per liter). The concentrations of both preservatives in the aliquots were equivalent to what is commonly utilized for preserving 24 h urine collections. After being thoroughly mixed, one aliquot was immediately frozen at −20 °C, while three aliquots were stored at different temperatures: at 4 °C, at room temperature (22 °C in the laboratory), and in an incubator (VWR Symphony, Radnor, PA, USA) at 40 °C to simulate extreme summer outdoor temperatures. The NP sample stored at −20 °C served as a control sample. After 24 h, 4.5 mL of each aliquot was transferred to a new tube and stored at −20 °C. Meanwhile, the remaining volume was incubated for an additional 24 h for a total of 48 h until being stored at −20 °C. The longer hold time of 48 h was included to simulate samples that are collected on a Saturday and returned by participants on the following Monday.

Prior to urine sample analysis, participants’ samples were pooled based on temperature (−20, 4, 22, and 40 °C), incubation time (24 and 48 h), preservative treatment (none, BA, thymol), and oral administration of PABA (PABA, no PABA), resulting in 42 analytical groups. The pooling of samples was performed to maximize the array of metabolites and potential for bacterial contamination in urine, while limiting the effect of between-subject variability on metabolite concentrations. 

### 4.3. Chemicals

BA was purchased from Sigma-Aldrich (St. Louis, MO, USA). Thymol was bought from Science Lab (Houston, TX, USA). Internal standards, L-glutamic acid-^13^C_5_ and sodium L-lactate-^13^C_3_, were purchased from Cambridge Isotope Laboratories, Inc. (Tewksbury, MA, USA). Methanol, isopropanol, and acetonitrile (ACN) (all HPLC-grade) were purchased from VWR International (Radnor, PA, USA). Ammonium acetate (NH_4_OAc) and ammonium hydroxide (NH_4_OH) solution were purchased from Sigma-Aldrich (St. Louis, MO, USA). Deionized water was provided by an in-house Milli-Q^®^ Water Purification System (Billerica, MA, USA). Phosphate-buffered saline (PBS) was purchased from GE Healthcare Life Sciences HyClone Laboratories (Chicago, IL, USA).

### 4.4. Urine Sample Preparation

The sample preparation protocol used here was modeled after that employed in previous studies [5,48,49,50]. Each urine sample (50 µL) was mixed with 500 µL MeOH and 50 µL internal standard solution, MeOH:H2O = 1:1 solution containing 1.81 mM L-lactate-^13^C_3_ and 142 µM L-glutamic acid-^13^C_5_, which was used to monitor the analytical variations during LC-MS/MS analysis thus ensure the precision and accuracy of the results. The mixtures were vortexed for 5 s and stored at −20 °C for 20 min to minimize enzymatic activity during protein precipitation, followed by centrifugation at 14,000 rpm for 10 min. Afterward, 450 µL of supernatant was transferred into a new 2 mL Eppendorf vial and dried in a CentriVap Concentrator at 37 °C for 120 min. The dried samples were then reconstituted in 150 µL of 40% PBS/60% acetonitrile (ACN) and vortexed for another 5 s. Following centrifugation at 14,000 rpm for 10 min, 100 µL of the supernatant was transferred into a LC vial for LC-MS analysis. The remaining 50 µL of supernatant from each sample were pooled together and used as the quality-control (QC) sample. The QC sample was analyzed once every 10 study samples.

### 4.5. LC-MS/MS Experiments

Targeted metabolic profiles (278 targeted metabolites) of urine samples were obtained by the LC-MS/MS method using an Agilent Technologies 1290 UPLC-6490 Triple Quadrupole MS system (Santa Clara, CA, USA). Separation by liquid chromatography was performed on an Xbridge^®^ BEH Amide column (2.5 μm, 2.1 × 150 mm; Waters, MA, USA) at 40 °C. The injection volume was set to 4 μL for positive ion mode and 10 μL for negative ion mode. The flow rate was 0.3 mL/min. The stock solution was 10 mM NH_4_OAc and 10 mM NH_4_OH in ACN. The mobile phase system was composed of solvents A (ACN: stock = 5:95) and B (ACN: stock = 95:5) for both positive and negative ionization modes. The LC gradient conditions were set as follows for both positive and negative ionization modes: after an initial 1.0 min isocratic elution of 10% A, the percentage of Solvent A was increased linearly to 60% at t = 11 min. After 4.5 min (t = 15.5 min), the percentage of A was reduced to 10% to prepare for the next injection. The total experimental time for each injection was 30 min.

The QQQ mass spectrometer was operated under the following conditions: the capillary voltage for both positive and negative ionization modes was 3.5 kV, the gas temperature was 175 °C, the nitrogen flow rate was 15 L/min, the nebulizer gas pressure was set to 30 psi, the sheath gas temperature was 225 °C, and the sheath gas flow rate was 11 L/min. 

### 4.6. Statistical Analysis

The extracted MRM peaks were integrated using QQQ Quantitative Analysis software (Version B.08.00, Agilent Technologies, Santa Clara, CA, USA). After integration, filtering was performed, and variables with coefficients of variation (CVs) in the QC data < 20% and with 80% signal intensities > 1000 in each urine sample batch (positive or negative) were retained, resulting in 158 reliably detected metabolites. We performed principal components analysis (PCA) using the SIMCA 13.0 software (Umetrics, Umeå, Sweden). The data were log10-transformed prior to PCA analysis. No data normalization was used. Volcano plots were obtained using Microsoft Office Excel 2013 (Redmond, WA, USA).

## Figures and Tables

**Table 1:** Urine storage conditions under investigation in both para-aminobenzoic acid (PABA) and No PABA groups.

Temperature	Storage Time	Preservative
Freezer (−20 °C) * Refrigerator (4 °C) Room temperature (22 °C) High temperature (40 °C)	- 24 h, 48 h 24 h, 48 h 24 h, 48 h	NP, BA, Thy NP, BA, Thy NP, BA, Thy NP, BA, Thy

* Urine samples with NP stored in the freezer were used as control samples. NP: No preservative; BA: Boric acid; Thy: Thymol.

**Table 2:** Effects of temperature and duration of storage on urine metabolites.

Temperature	NP Urine Sample Clustered with Control	
	24 h	48 h
Refrigerator (4 °C) Room temperature (22 °C) High temperature (40 °C)	√ √ ×	√ × ×

√: clustered with control (−20 °C); ×: further apart from control (−20 °C).

**Table 3:** Significantly altered metabolites caused by high temperature (40 °C) in urine samples with no preservative or preserved with thymol in the PABA group.

Preservative.	Metabolite	*p*-Value	Fold Change (−20 °C, 4 °C)/(40 °C)	Metabolite Changes *
No preservative	Sucrose	0.004	13.723	Decreased
	Trehalose	0.006	11.162	Decreased
	Adenosine	0.005	5.895	Decreased
	Acetylglucosamine	0.005	3.492	Decreased
	Sorbitol	0.027	3.188	Decreased
	L-Alloisoleucine/Leucine/Norleucine	0.050	2.833	Decreased
	Lactose	0.007	2.620	Decreased
	Fructose	0.018	2.300	Decreased
	Tyrosine	0.003	2.224	Decreased
	Isoleucine	0.000	2.068	Decreased
	2-Deoxyadenosine	0.019	2.066	Decreased
	5-Aminolevulinic acid	0.017	0.396	Increased
	Adenine	0.001	0.312	Increased
	Cytidine	0.003	0.309	Increased
	Lactate	0.039	0.173	Increased
	3-Phenyllactic acid	0.023	0.171	Increased
Thymol	-	-	-	-

* Metabolite changes in 40 °C urine samples (24 and 48 h) compared to −20 and 4 °C urine samples (24 and 48 h).

**Table 4:** Significantly altered metabolites at high temperature (40 °C) in urine samples with no preservative or preserved with thymol in the No PABA group.

Preservative	Metabolite	*p*-Value	Fold Change (−20 °C, 4 °C)/(40 °C)	Metabolite Changes *
No preservative	Methionine	0.033	5.715	Decreased
	Adenosine	0.021	2.740	Decreased
	Fructose	0.012	2.345	Decreased
	Lactate	0.011	0.321	Increased
	3-Phenyllactic acid	0.004	0.212	Increased
Thymol	Lactose	0.041	2.066	Decreased
	Adenine	0.048	0.475	Increased
	Hydroxyproline	0.004	0.323	Increased

* Metabolite changes in 40 °C urine samples (24 and 48 h) compared to −20 and 4 °C urine samples (24 and 48 h).

**Table 5:** Guidelines for urine sample storage for metabolomics analysis.

Temperature	Storage Period	Preservative
4 °C	24 h	-
	48 h	-
22 °C	24 h 48 h	-
		Thymol
40 °C	24 h	Thymol
	48 h	Thymol

## Supplementary Materials

The following are available online at https://www.mdpi.com/2218-1989/9/10/203/s1, Figure S1: Volcano plots of no preservative versus preservatives for urine samples stored at −20 °C, 4 ° for 24-h/48-h, and at 22 ° for 24-h, Figure S2: Chromatograms by targeted liquid chromatography tandem mass spectrometry (LC-MS/MS)-based metabolomics, Table S1: Significantly altered metabolites caused by boric acid and thymol compared to urine samples with No Preservative stored at −20 °, 4 ° for 24-h/48-h, and at 22 ° for 24-h in the PABA and No PABA Group.
