# Supplementary material for: Influence of Storage Conditions and Preservatives on Metabolite Fingerprints in Urine
Source: Metabolites. 2019 Sep 27;9(10):203. doi: 10.3390/metabo9100203 (PMC6836253; doi:10.3390/metabo9100203)
Supplement: Supplementary file 1 [file metabolites-09-00203-s001.pdf]

Supplementary Materials

Influence of Storage Conditions and Preservatives on Metabolite Fingerprints in Urine

Xinchen Wang[1,2+], Haiwei Gu[2+], Susana A. Palma-Duran[2], Andres Fierro[2], Paniz Jasbi[2], Xiaojian Shi[2], William Bresette[2], Natasha Tasevska[2]*

[1]Jiangxi Key Laboratory for Mass Spectrometry and Instrumentation, East China University of Technology, Nanchang, Jiangxi 330013, China

[2]College of Health Solutions, Arizona State University, Phoenix, AZ 85004, USA

[+]These authors contributed equally to the study

(A)

(B)

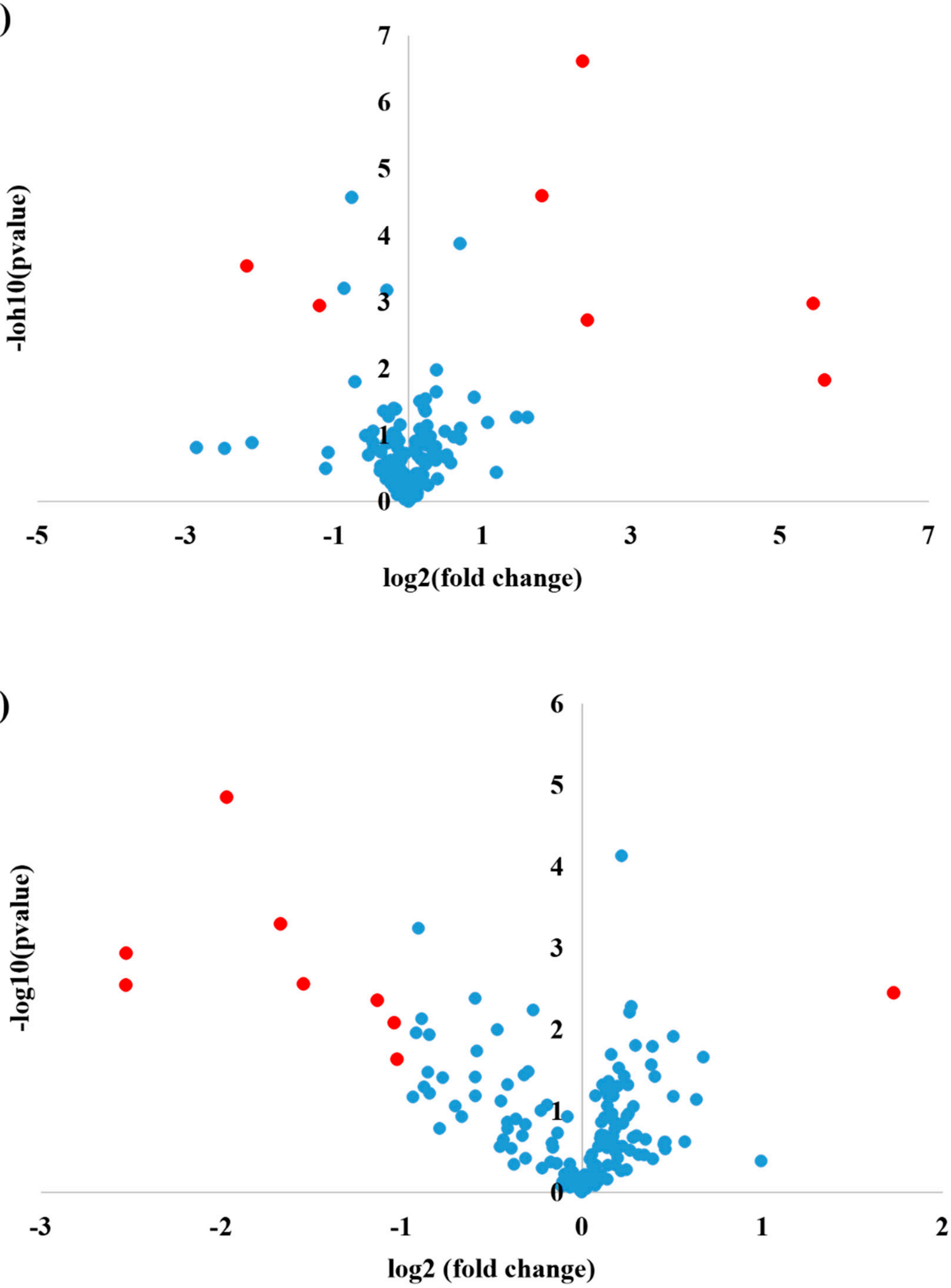
-loh10(pvalue)
log2(fold change)
7
6
5
4
3
2
1
0
-5
-3
-1
1
3
5
7
-log10(pvalue)
log2 (fold change)
6
5
4
3
2
1
0
-3
-2
-1
0
1
2

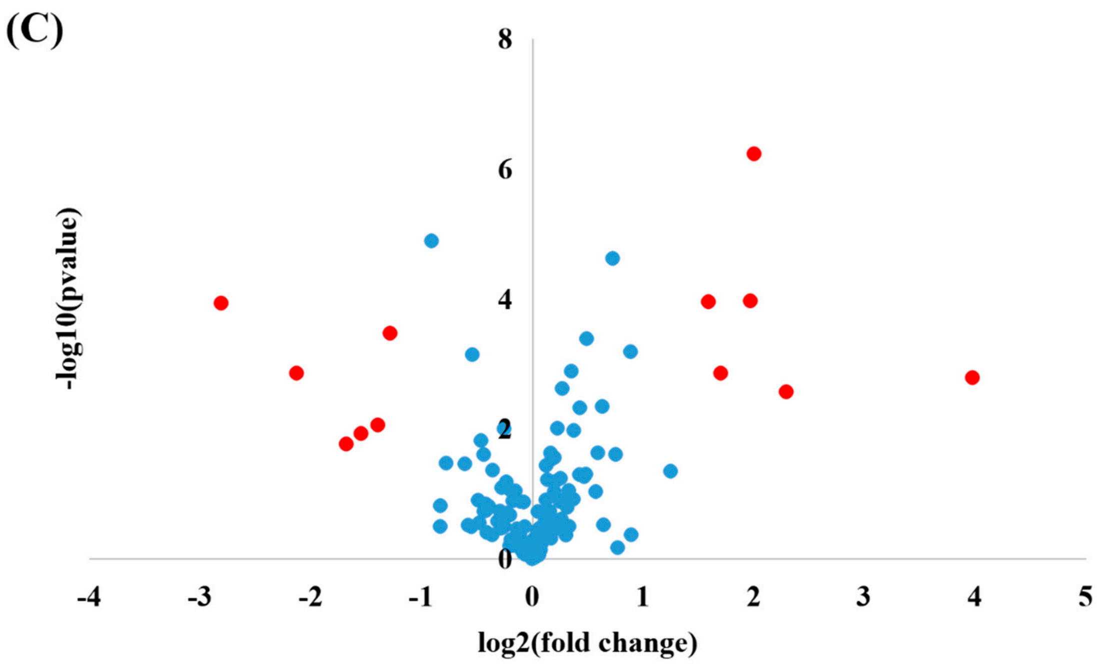

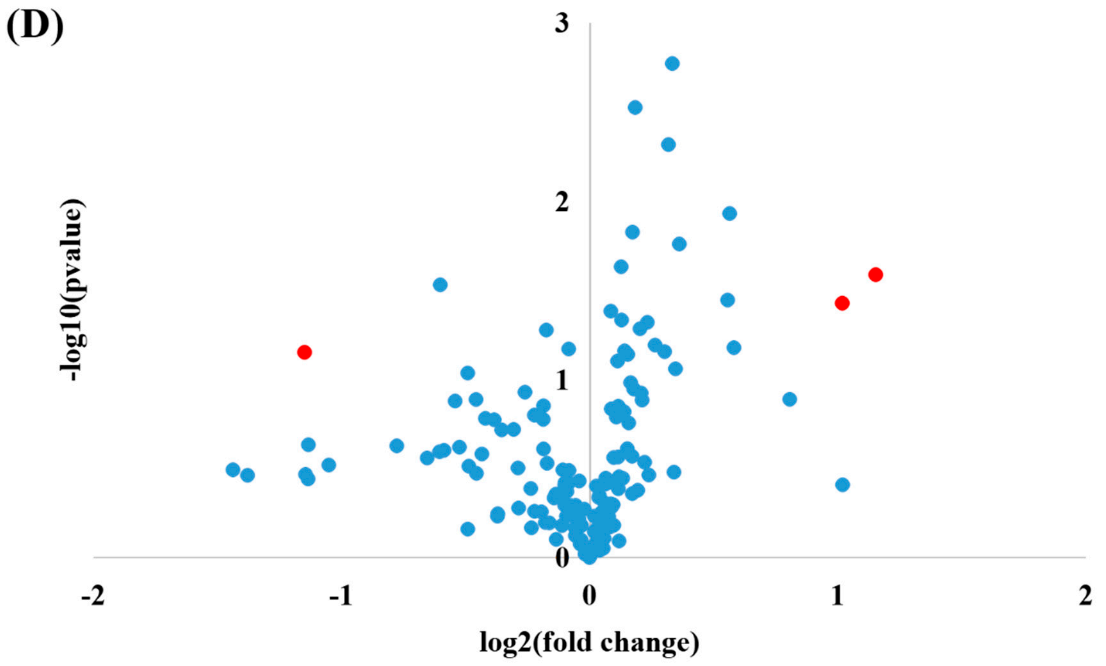

Supplementary Figure 1. Volcano plots of no preservative versus preservatives for urine samples stored at -20˚C, 4˚C for 24-h/48-h, and at 22˚C for 24-h **in the PABA group:** (A) no preservative versus boric acid, (B) no preservative vs. thymol; and **in the No PABA group:** (C) no preservative vs. boric acid, (D) no preservative vs. thymol. Red dots indicate metabolites with p-value <0.05 and fold change >2 or <0.5.

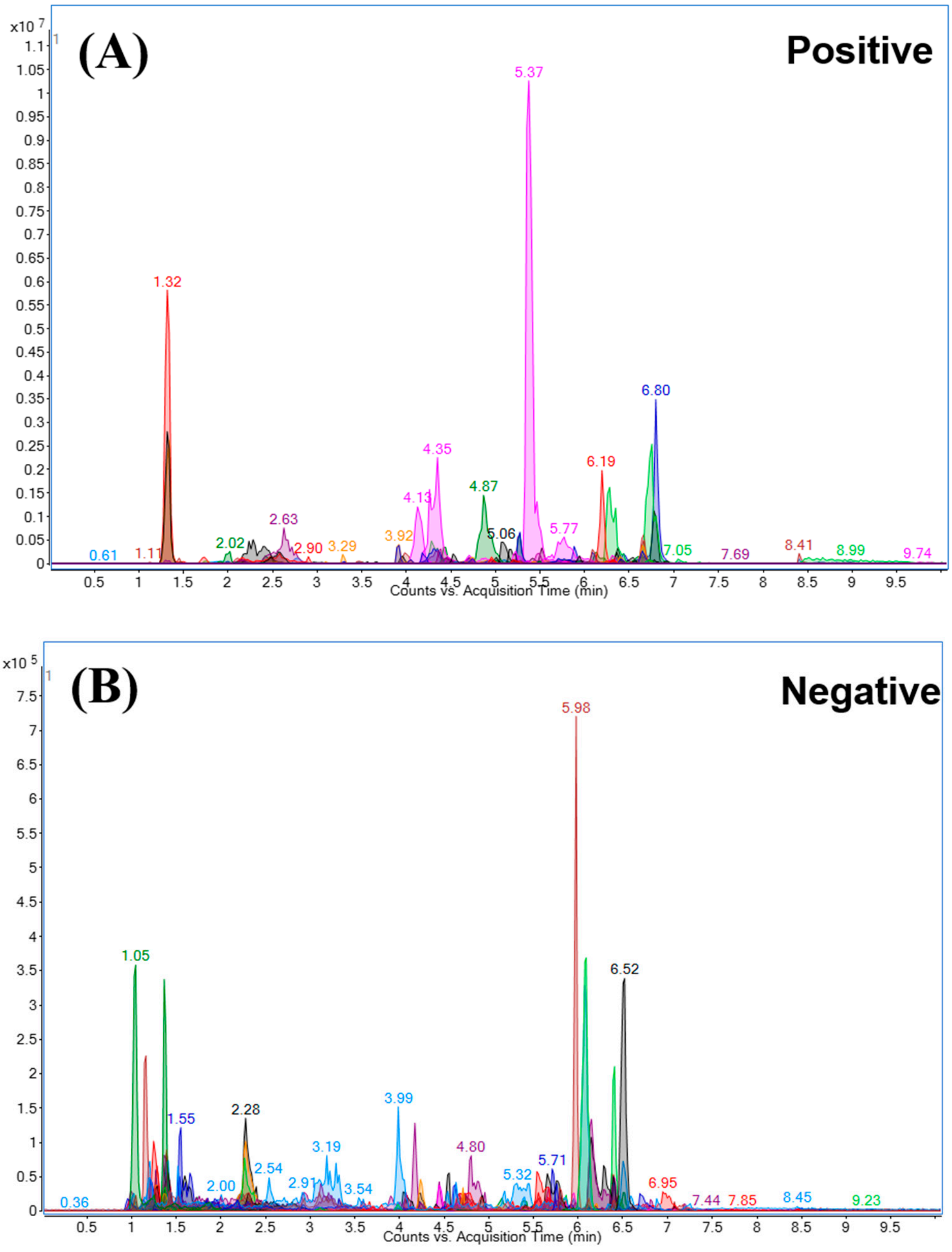

Supplementary Figure 2. Chromatograms by targeted liquid chromatography tandem mass spectrometry (LC-MS/MS)-based metabolomics in (A) positive ion mode and (B) negative ion mode.

Supplementary Table 1. Significantly altered metabolites caused by boric acid and thymol compared to urine samples with No Preservative stored at -20˚C, 4˚C for 24-h/48-h, and at 22˚C for 24-h in the PABA and No PABA Group

Metabolite	P-value	Fold Change (NP)/(BA)	Metabolite changes*
**PABA group**			
Boric Acid vs. No Preservative			
Pyridoxine	0.015	49.600	Decreased
2-Deoxyadenosine	0.001	38.289	Decreased
D-Galacturonic acid	0.002	5.054	Decreased
belta-Hydroxyisovaleric acid	<0.001	4.787	Decreased
Xanthurenic acid	<0.001	3.631	Decreased
Adenosine	<0.001	0.205	Increased
GA3P	0.001	0.428	Increased
Thymol vs. No Preservative			
3-Methyladipic acid	<0.001	3.708	Decreased
3-Hexenedioic acid	<0.001	0.176	Increased
Isovaleric acid	<0.001	0.232	Increased
Agmatine	<0.001	0.322	Increased
Dopamine	0.015	0.444	Increased
Citrulline	0.003	0.454	Increased
Alpha-KG/Adipic acid	0.016	0.465	Increased
2-Methylglutaric acid	0.030	0.473	Increased
Glutamic acid	0.044	0.494	Increased
**No PABA Group**			
Boric Acid vs. No Preservative			
2-Deoxyadenosine	<0.001	13.129	Decreased
Pyridoxine	0.003	4.909	Decreased
Adenine	0.001	3.257	Decreased
Xanthurenic acid	<0.001	4.344	Decreased
Belta-Hydroxyisovaleric acid	<0.001	3.969	Decreased

D-Galacturonic acid	<0.001	3.017	Decreased
Isovaleric acid	0.001	0.229	Increased
Adenosine	0.001	0.168	Increased
Alpha-KG/Adipic acid	<0.001	0.411	Increased
Dopamine	0.002	0.263	Increased
Pentadecanoic acid	0.002	0.380	Increased
Phenylglyoxylic acid	0.005	0.394	Increased
Thymol vs. No Preservative			
Tryptamine	0.009	2.013	Decreased
Anthranilic acid	0.026	2.225	Decreased
Lauric acid	0.031	0.480	Increased
